# Genome-Wide Identification of the *Early Flowering 4* (*ELF4*) Gene Family in Cotton and Silent GhELF4-1 and GhEFL3-6 Decreased Cotton Stress Resistance

**DOI:** 10.3389/fgene.2021.686852

**Published:** 2021-07-06

**Authors:** Miaomiao Tian, Aimin Wu, Meng Zhang, Jingjing Zhang, Hengling Wei, Xu Yang, Liang Ma, Jianhua Lu, Xiaokang Fu, Hantao Wang, Shuxun Yu

**Affiliations:** ^1^Engineering Research Centre of Cotton, Ministry of Education, College of Agriculture, Xinjiang Agricultural University, Ürümqi, China; ^2^State Key Laboratory of Cotton Biology, Institute of Cotton Research, Chinese Academy of Agricultural Sciences, Anyang, China

**Keywords:** ELF4, cotton, fiber, expression patterns, gene silencing

## Abstract

The early flowering 4 (ELF4) family members play multiple roles in the physiological development of plants. *ELF4s* participated in the plant biological clock’s regulation process, photoperiod, hypocotyl elongation, and flowering time. However, the function in the *ELF4s* gene is barely known. In this study, 11, 12, 21, and 22 *ELF4* genes were identified from the genomes of *Gossypium arboreum*, *Gossypium raimondii*, *Gossypium hirsutum*, and *Gossypium barbadense*, respectively. There *ELF4s* genes were classified into four subfamilies, and members from the same subfamily show relatively conservative gene structures. The results of gene chromosome location and gene duplication revealed that segmental duplication promotes gene expansion, and the Ka/Ks indicated that the ELF4 gene family has undergone purification selection during long-term evolution. Spatio-temporal expression patterns and qRT-PCR showed that GhELF4 genes were mainly related to flower, leaf, and fiber development. *Cis-*acting elements analysis and qRT-PCR showed that GhELF4 genes might be involved in the regulation of abscisic acid (ABA) or light pathways. Silencing of *GhELF4-1* and *GhEFL3-6* significantly affected the height of cotton seedlings and reduced the resistance of cotton. The identification and functional analysis of ELF4 genes in upland cotton provide more candidate genes for genetic modification.

## Introduction

The early flowering 4 (ELF4) family belongs to a small and highly conserved gene family, discovered and named by Doyle (Doyle et al., 2002). The conserved domain of the ELF4 gene family is DUF1313, which is a highly conserved domain. At present, it belongs to the domain of unknown function, and few researches have been reported in this area. The ELF4s gene family has been identified in five members of *Arabidopsis* (Doyle et al., 2002; Khanna et al., 2003; Kikis et al., 2005; Harriet et al., 2007; Kolmos and Davis, 2007), three of *Doritaenopsis hybrid* (Chen et al., 2015b), and nine of *Glycine max* (Juliana et al., 2017). Studies have shown that *ELF4* is a central gene in the circadian clock, which is involved in photoperiod perception, circadian regulation, signal transmission in the early flowering of *Arabidopsis*, promotion of seedling de-etiolation, and hypocotyl growth of *Arabidopsis* (Doyle et al., 2002; Khanna et al., 2003; Harriet et al., 2007; Kolmos and Davis, 2007; Nusinow et al., 2011).

The plant circadian clock is an essential method for plants to control their everyday life activities (Li and Zhang, 2015). Its normal operation of circadian clock can ensure the good growth in plants. Disturbance of its functions causes serious damage to the growth of plants and directly affects their normal physiological metabolism, which leads to a reduction in crop production and income (McClung, 2006; Farooq et al., 2018). Furthermore, the ELF4 is a component of the central circadian clock associated 1 (CCA1)/late elongated hypocotyl (LHY) – timing of CAB expression 1 (TOC1) feedback loop in the circadian clock (Doyle et al., 2002), which is a central gene to improve clock accuracy (Doyle et al., 2002). In *Arabidopsis*, deletion of the *AtELF4* gene leads to an impaired photoperiod response, and circadian dysfunction (imprecision and arrhythmicity), and very low amplitude of the light-induced clock gene *CCA1* (Doyle et al., 2002).

Regarding hypocotyl elongation, light and the circadian clock interact to consolidate the phase of hypocotyl cell elongation to dawn under diurnal cycles in *Arabidopsis*. In addition, studies have shown that the transcription repressor evening complex (EC), composed of ELF4, lux arrhythmo (LUX) and early flowering 3 (ELF3), is the core component of the plant circadian clock (Silva et al., 2020). EC can inhibit the expression of *phytochrome interacting factor 4* (*PIF4*) and *PIF5* expression in the evening, leading to hypocotyl elongation (Nusinow et al., 2011). Furthermore, it was found that the hypocotyl of elf4-1 mutant is longer than the wild type in *Arabidopsis* (Chen et al., 2015a). *DhEFL2*, *3*, and *4* of *Doritaenopsis hybrid* ELF4 family gene can rescue *elf4* mutant growth phenotype (Chen et al., 2015a).

In the flowering study, the expression of *AtCO* and *AtFT* decreased after *GmELF4* gene of soybeans overexpression resulted in delayed flowering (Juliana et al., 2017). *Doritaenopsis hybrid* genes *DhEFL2*, *3*, and *4* are also related to flowering regulation (Chen et al., 2015a). And studies have found that *ELF4* inhibits the expression of *CO*, causing the *elf4-1* mutant to show early flowering (Doyle et al., 2002).

ELF4 functions as a signaling intermediate in phy-regulated gene expression involved in promotion of seedling de-etiolation (Kikis et al., 2005). The *elf4* mutant seedlings are impaired in phyB-mediated seedling de-etiolation (Kikis et al., 2005). ELF4 is also very important in the process of seedling de-etiolation. It is a phytochrome-regulated component of a negative feedback loop involving the central oscillator components CCA1 and LHY, and it is necessary for light-induced expression of both CCA1 and LHY.

Globally, cotton is one of the necessary cash crops and is grown in about 100 countries/regions (Farooq et al., 2018). It is crucial to study the physiological mechanism of cotton for its growth and development and to ensure a high yield. Thus far, the roles of ELF4s in the light-responsive molecular pathway, circadian clock and abscisic acid (ABA) responsive pathway of cotton are still relatively unknown. To better to understand the potential mechanism ELF4 gene family in cotton, a comprehensive analysis of ELF4s in four cotton species was performed. In addition, we also explored the phylogenetic relationship, gene structure, amplification mode, *cis-*acting elements, and expression mode of the ELF4 family. The results of this study will help us understand the structure, expression, and potential functions of the ELF4 gene family in cotton and provide a basis for studying the biological rhythm of the ELF4 gene family in cotton.

## Materials and Methods

### Identification of Members of the ELF4 Gene Family in Cotton and Other Species

The genome data of *G. arboreum* (Du et al., 2018), *G. raimondii* (Paterson et al., 2012), *G. hirsutum* (Wang et al., 2019), and *G. barbadense* (Wang et al., 2019) were from CottonGen website download (Sun et al., 2019). The protein sequences of *Arabidopsis thaliana*, *Populus trichocarpa*, *Glycine max*, *Theobroma cacao*, and *Oryza sativa* were downloaded from the Phytozome website. The Hidden Markov Model (Potter et al., 2018) profile of the ELF4 conserved domain (Pfam07011) was obtained from the Pfam database (Finn et al., 2016). Using the hmmsearch program of the HMMER 3.0 software (Potter et al., 2018), the ELF4 conserved domains of cotton and other species were queried with HMM files, and the *E* values threshold was 1.0 E-30 (Sun et al., 2018). In addition, the online SMART and NCBI-CDD website were used to confirm the conserved domains of all ELF4 protein sequences to ensure that the selected gene had ELF4 conserved domain (Letunic et al., 2015).

Predict the physicochemical properties of ELF4s family proteins through ExPASy webpage, and used SMART to predict the subcellular localization of ELF4s.

### Sequence Alignment and Phylogenetic Analysis of the ELF4 Gene Family

All predicted ELF4s gene sequences were multiple-aligned by ClustalX 2.0 (Wu et al., 2019). An unrooted phylogenetic tree was generated using the neighbor-joining (Zhao et al., 2018) method and the amino acid p-distance model in MEGA 6.0. Bootstrap resamplings (1000) were used to assess interior branches’ reliability (Sun et al., 2019; Silva et al., 2020).

### Chromosome Location and Collinearity Analysis

MapChart software was used to display the location of genes on chromosomes (Voorrips, 2002). The genomic files of the cotton species were sequence aligned with the Basic Local Alignment Search Tool (BLAST) for collinear analysis, with a cut-off *E* value of 1 × 10^−5^ (Sun et al., 2019). MCScanX software was used to perform the collinearity analysis based on the results of BLAST (Wang et al., 2012). Gene replication was confirmed according to the following conditions: the coverage of the aligned sequence >75%; the similarity of the aligned sequence >75%, and the replication gene pairs of the ELF4 gene family are screened. To gain greater insight into the expansion pattern of in this ELF4 gene family, we identified tandem duplicated gene pairs and segment duplicated gene pairs based on the gene locus, and tightly linked genes on the same chromosome were considered tandem duplications (Wei et al., 2007; Zhang et al., 2011).

The results were visualized using the TBtools software or MCScanX software (Chen et al., 2020).

### Gene Structure and Conserved Motif Analysis

The online tool WebLogo-online logo sequence generator was used to obtain the sequence logo of the DUF1313 (Pfam07011) domain (Crooks et al., 2004). Visualize the exon–intron structure of ELF4 genes through the Gene Structure Display Server (GSDS). The conserved motifs of the GhELF4 protein sequence were analyzed using the MEME program (Bailey et al., 2006; Wu et al., 2019).

### Evolutionary Selective Pressure

Non-synonymous (Ka) and synonymous substitution (Ks) rates were calculated using the DnaSp V5.0 software. The Ka/Ks ratio was assessed to determine the molecular evolutionary rates of each gene pair (Chen et al., 2020).

### Analysis of *Cis*-Elements of Upstream Sequences

To predict the *cis-*acting elements of the promoters of GhELF4 genes, the 2,000 bp DNA sequence upstream of the codons (ATG) of GhELF4 genes was extracted from the genome database and analyzed on the Plant CARE website (Lescot, 2002).

### Expression Analysis of GhELF4s Based on Transcriptome Data

The RNA-seq data were downloaded from the NCBI Sequence Read Archive (SRA: PRJNA248163) and the CottonFGD website. RNA-seq expression was analyzed using TopHat2 (Kim et al., 2013) and cufflinks (Trapnell et al., 2010). The fragments per kilobase million (FPKM) values denoting the expression levels of *ELF4* genes were isolated from a comprehensive profile of the TM-1 transcriptome data (Trapnell et al., 2010). A heat-map analysis was performed using TBtools program (Trapnell et al., 2010). The GhELF4s with an FPKM > 1 were used for further expression analysis.

### Plant Materials and Treatments

The *G. hirsutum* cultivars TM-1 were field grown in Anyang, Henan province, China. The roots, stems, bud, leaves (three-leaf-period cotton plants), and flowers of TM-1 were harvested for RNA extraction. The fibers of TM-1 were separated from the ovules 0, 5, 10, 15, 20, and 25 days postanthesis (DPA) for RNA extraction.

TM-1 was grown in a climate-controlled greenhouse (light/dark cycle: 16/8 h at 28°C. The lights were turned on in sequence from 6:00 to 6:30, and the lights were turned off in the same order from 22:00 to 22:30). When the third true leaf was spreading, the tender buds were taken. The whole buds were collected at 5:00, 6:00, 6:30, 7:00, 15:00, 22:00, 22:30, and 23:00. Each sample was repeated in three groups and all samples were immediately frozen in liquid nitrogen and stored at −80°C. The TM-1 seedlings of the flat-leaf period of the third true leaf were sprayed with 100 μmol/L ABA and water. Cottonleaf samples were collected at 3 h, 6 h, 9 h, 12 h, and 24 h after treatment. Three samples were taken at each time and all samples were rapidly frozen in liquid nitrogen and stored at −80°C.

### RNA Extraction and qRT-PCR Analysis

The Tiangen RNAprep Pure Plant kit (Tiangen, China) was used to extract the total RNA in the collected samples. First-strand cDNA was synthesized via reverse transcription of 1 μg of the total RNA using the PrimeScript RT Reagent kit (Takara, Japan). Used Oligo 7.0 software or Primer5.0 to design specific primers for qRT-PCR.

The internal control used the histone-3 gene (GenBank accession no. AF094716) (Tu et al., 2007; Sun et al., 2018). The qRT-PCR experiments were performed using SYBR Premix Ex Taq (Takara) on an ABI 7500 real-time PCR system (Applied Biosystems, United States) with three replicates (Sun et al., 2018). The relative expression levels of GhELF4s was calculated by 2^−ΔΔ^^CT^ method, and statistical analysis was performed using *t*-tests (Livak and Schmittgen, 2001).

### Construction of Virus-Induced Gene Silencing Vectors of GhELF4-1 and GhEFL3-6 in Cotton

Virus-induced gene silencing (VIGS) is an RNA-mediated gene silencing after transcription that depends on plants’ defense mechanism against viruses (Senthil-Kumar and Mysore, 2011). A segment of the target gene *GhELF4-1* (*Ghir_A06G017580.1*) with a length of about 300 bps was ligated into the shuttle plasmid pYL-156 to construct the vector pYL-156-GhELF4-1. The same method is used to construct vector pYL-156-GhEFL3-6 with *GhEFL3-6* (*Ghir_D06G008170.1*) gene. The positive control vector (pYL-156-CLA1), the negative control (pYL-156), the auxiliary plasmid (pYL-192) and the constructed vector containing the target gene fragments were transformed into *Agrobacterium tumefaciens* strain. In the period when the cotyledons are flat, the method of infecting cotton cotyledons with Agrobacterium is used (Gao and Shan, 2013). After 2 weeks, the total RNA of cotton leave was extracted, and the gene silencing was detected by qRT-PCR technology.

Peroxidase (POD) activity and catalase (Silva et al., 2020) activity were measured for the intrinsic stress resistance indexes of VIGS cotton plants. Take 0.1 g of fresh cotton leaves to determine the activity of CAT or POD enzymes, and take three samples from each plant for biological repetition. CAT and POD activity were measured using the kit developed by Solarbio Biology Co., Ltd., and the specific operation steps were guided according to the operating instructions.

## Results

### Identification of ELF4 Family Genes in Different Species

Based on the HMM model of the ELF4 specific conserved domain constructed by the DUF1313 (Pfam07011) protein sequence, a total of 11, 12, 21, and 22 ELF4 genes were identified from *G. arboreum*, *G. raimondii*, *G. hirsutum*, and *G. barbadense*, respectively (Supplementary Table 1). All these putative genes were detected to contain the typical DUF1313 domain of the ELF4 gene family in the NCBI-CDD and SMART databases.

According to their locations on the chromosomes, the family members of the *G. hirsutum* were designated *GhELF4-1* to *GhELF4-5*, *GhEFL4-1* to *GhEFL4-5*, *GhEFL3-1* to *GhEFL3-9*, and *GhEFL1-1* to *GhEFL1-2*, and the same applied to the naming of the other species.

The lengths of the putative GhELF4 proteins varied from 105 amino acids (aa) (GaELF4-3) to 127 aa (GaEFL3-3); while those of GhELF4s ranged from 105 aa (GhELF4-5 and GhELF4-3) to 134 aa (GhEFL1-2); those of GbELF4s varied from 105 aa (GbELF4-3) to 141 aa (GbEFL3-8); and those of GrELF4s varied from 73 aa (GrELF4-1) to 178 aa (GrEFL1). In cotton, the MW values ranged from 8514.23 to 136817.27. The theoretical isoelectric points of the ELF4 protein ranged from 4.8024 to 10.3931. For a detailed analysis of the other proteins, such as subcellular localization and hydrophilicity, see Supplementary Table 1.

### Chromosomal Distribution of ELF4s in 4 Cotton Species

**FIGURE 1:**
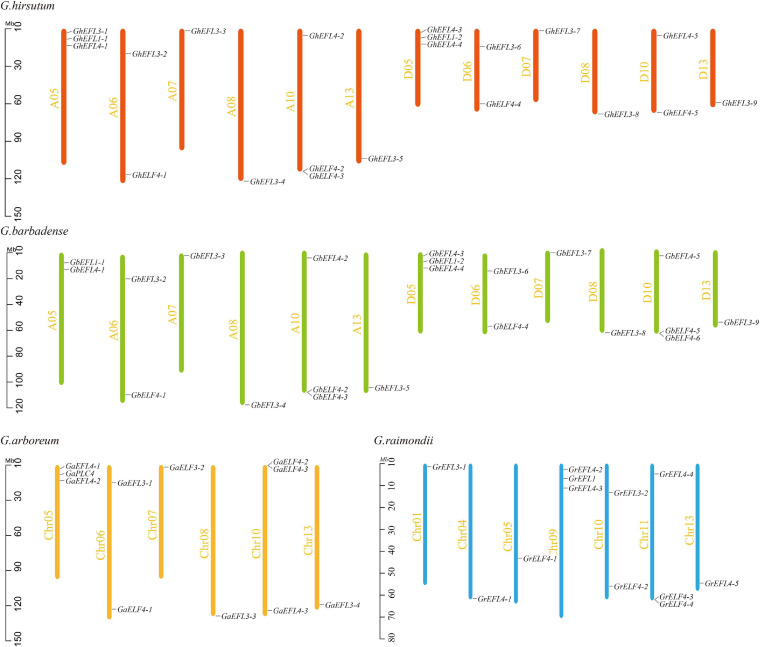
Chromosomal distribution of ELF4s in *Gossypium raimondii*, *G. barbadense*, *G. arboreum*, and *G. hirsutum*. The scale represents megabases (Mb). The chromosome numbers are indicated on the left of each vertical bar.

The location information is listed in Supplementary Table 2. The chromosome location of each gene and the number of target genes for each chromosome are shown in Figure 1. In *G. hirsutum*, 21 GhELF4s were unevenly anchored on 12 chromosomes. There were three chromosomes (A05, A10, and D05) that contained three genes, three chromosomes (A06, D06, and D10) that contained two genes, and the other chromosomes only contained one gene. The distribution of the GbELF4s in the chromosome was very similar to that of GhELF4s, except one ELF4 gene was located on the scaffold. In *G. arboreum*, 11 GaELF4s were located on six chromosomes. Both Chr05 and Chr10 contained the most GaELF4s (three genes). There were two genes located on Chr06 chromosomes. Furthermore, Chr07, Chr08, and Chr13 contained one gene. In *G. raimondii*, 12 GrELF4s were unevenly anchored on seven chromosomes (Chr01, Chr04, Chr05, Chr09, Chr10, Chr11, and Chr13). There were two chromosomes (Chr09 and Chr11) that contained three genes, one chromosome (Chr10) that contained two genes, and the other chromosomes only contained one gene.

According to the comparison of the ELF4 genes distribution of different cotton varieties, the ELF4 gene family has a conservative chromosome distribution and a number of conserved genes in cotton.

### Phylogenetic Analysis of the ELF4 Family

**FIGURE 2:**
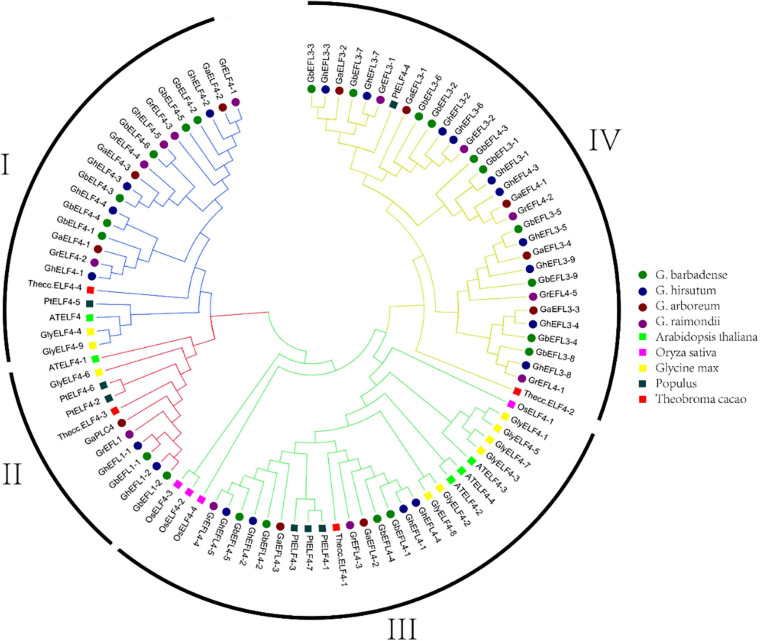
Phylogenetic tree of ELF4s. The 95 ELF4 protein sequences of *G. raimondii*, *G. arboreum*, *G. hirsutum*, *G. barbadense*, *A. thaliana*, *Populus trichocarpa*, *Oryza sativa*, *Theobroma cacao*, and *Glycine max* were aligned with ClustalX 2.0, and the phylogenetic tree was generated.

On the basis of the conserved ELF4 conserved domain (Pfam07011) and SMART analyses, 12 ELF4s in *G. raimondii*, 11 in *G. arboretum*, 21 in *G. hirsutum*, 22 in *G. barbadense*, 9 in *Glycine max*, 7 in *Populus*, 4 in *Theobroma cacao*, 4 in *Oryza sativa*, and 5 in *Arabidopsis thaliana* were identified. The phylogenetic tree classified the ELF4 family into four major groups, I–IV (Deng et al., 2014), and the name of each subgroup was assigned according to previous results in *Arabidopsis* (Song et al., 2009; Chen et al., 2015a). As shown in Figure 2, group IV was the largest subgroup, containing 32 ELF4s. There were 23 ELF4 genes in group I, 11 in group II, and 29 in group III.

### Gene Duplication Events of ELF4s

Studies have shown that gene replication events often accompany the evolution of plant genomes and play an important role in the expansion of gene families. In order to explore the gene expansion mechanism of the ELF4s family in the evolution from diploid to tetraploid, we analyzed the gene replication events in GhELF4s, GaELF4s, and GrELF4s.

**FIGURE 3:**
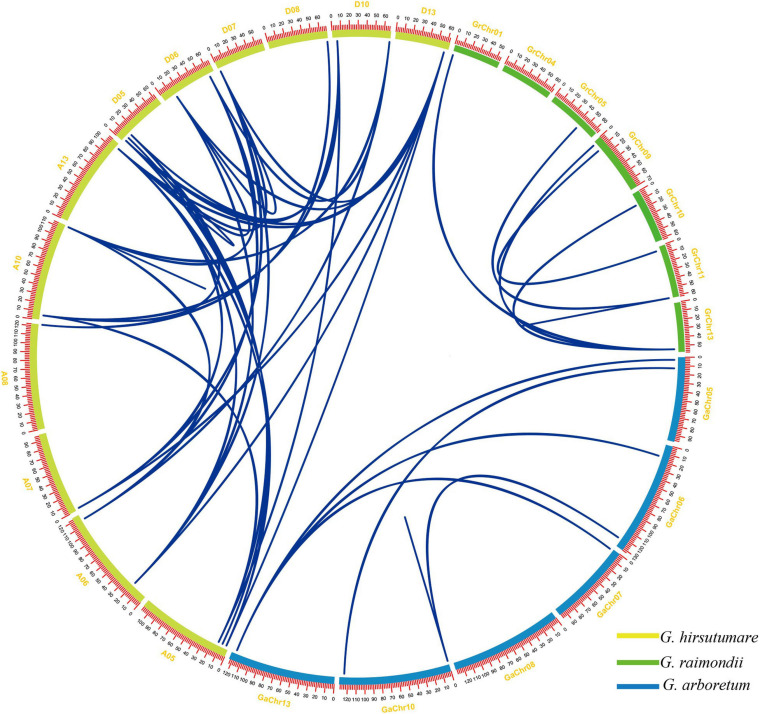
The gene pairs of segment duplication in *GrELF4s*, *GaELF4s*, and *GhELF4s.* The chromosomes of *G. raimondii*, *G. arboretum*, and *G. hirsutum* are filled with green, blue, and yellow, respectively. Gene pairs involved in segment duplication are linked by a line. The scale on the circle is in megabases (Mb).

In this study, as shown in Figure 3 and Supplementary Table 3, **52** pairs of gene duplication gene pairs were identified in *G. hirsutum*, *G. raimondii*, and *G. arboretum* (Supplementary Table 4). *G. arboretum*, *G. raimondii*, and *G. hirsutum* each have one tandem duplication gene pair (Supplementary Table 3), while 49 other pairs represented segmental duplication events; these pairs are shown in Figure 3. The Ka/Ks was calculated to assess the selection pressure of these homoeologous gene pairs. The Ka/Ks values of all the 52 gene pairs were less than 1 (Supplementary Table 3). The results showed that these ELF4s homoeologous gene pairs underwent purification selection after the gene duplication event.

### Conserved Sequence and Structure Analysis of ELF4s

Using the online tool WebLogo to construct sequence logos from the predicted ELF4s of the *G. arboreum*, *G. raimondii*, *G. hirsutum*, *G. barbadense*, *A. thaliana*, and *Populus trichocarpa* domain (Supplementary Figure 2), the results show that the DUF1313 domain of ELF4s was conserved in multiple species. The conserved domains of ELF4 proteins in four cotton species were analyzed using the conserved domain database of NCBI (Short name: DUF1313) (Supplementary Figure 1). Each of the 66 genes contained a DUF1313 domain. From Supplementary Figure 1, we can clearly see that there is a special gene, *GaPLC4* (*Ga05G0937*), which contains five domains, including DUF1313, PLN092952 superfamily, DUF4793, DUF4792, and zf-C3HC4_3.

**FIGURE 4:**
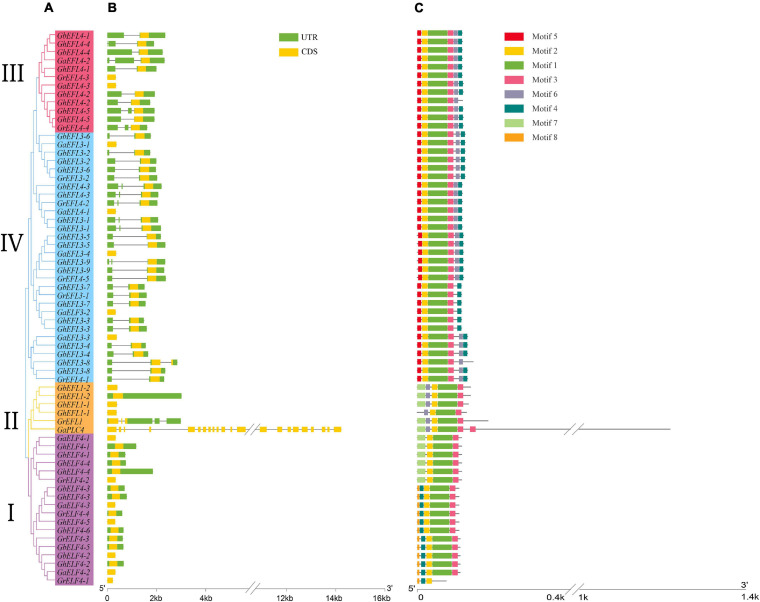
The gene structure and conserved protein motif of four cotton species ELF4. **(A)** The phylogenetic tree analysis of the ELF4 gene. **(B)** Analysis of exons and intron in ELF4 Genes. **(C)** The predicted ELF4 genes motif.

In order to understand the structural features of ELF4s, their gene exon–intron structures were analyzed using the online GSDS program. The gene structures of 66 ELF4 genes in cotton were analyzed, and four subfamilies were shown according to the phylogenetic tree (Figure 4A). The exon–intron structures of ELF4s are shown in Figure 4B. The number of ELF4 exons ranged from 1 to 22, but most contained less than five exons, except *GaPLC4*. The exon–intron structure of group I was more conserved than that of the other groups. Through comparative analysis of the gene structure (Figure 4B) and gene evolution tree (Figure 4A), it was found that the closer the genetic relationship, the more similar the gene structure and the number of introns. The positions of the conserved domains of the four cottons are detailed in Supplementary Table 4.

The MEME program was used for further analysis of conserved motifs. Eight conserved motifs were predicted and sequence logos of eight motifs were obtained (Figure 4C). In Figure 4C, ELF4s in the same group display a similar motif composition, which further supports the group classification result. Figure 4 shows that the exon–intron structure and motif distribution differed among different groups, while on the same branches, they were highly conserved.

### Analysis of *Cis*-Acting Elements in GhELF4 Genes Promoter Regions

To identify the potential *cis-*acting elements that may be involved in abiotic or biotic stress responses in the putative promoter region of the GhELF4 genes, the 2,000 bp upstream sequences of the 21 GhELF4 genes was extracted from the genome sequence.

**FIGURE 5:**
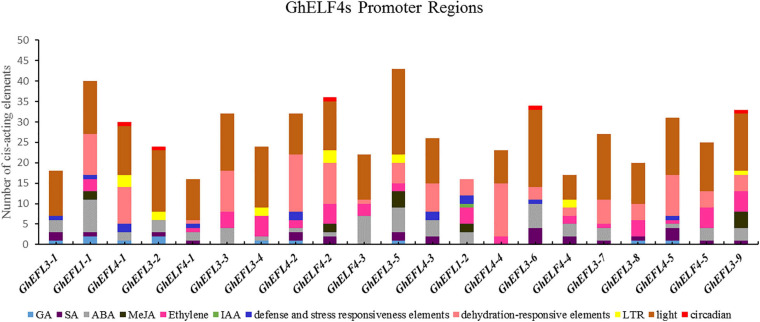
The *cis*-acting elements of stress-related and plant hormone response in the GhELF4 genes promoter regions. The proportion of each type of related *cis*-acting elements in 21 *GhELF4* genes.

The statistical results for the *cis-*acting elements indicated that many phytohormones-responsive elements were located on the promote regions of Figure 5, including abscisic acid (Chaban et al., 2003) responsive elements (ABRE) (Chaban et al., 2003), ethylene responsive element, gibberellin (GA) responsive element (P-box, TATC-box), jasmonate (MeJA) responsive element (T/G-box), the auxin (IAA) responsive element (TGA-element), and the salicylic acid responsive element (TCA-element). And some cis-acting elements in the promote regions of the GhELF4s were related to abiotic stress included low-temperature responsive element (LTR), dehydration-responsive elements (MYB and MYC), *cis-*acting element involved in defense and stress responsiveness (TC-rich repeats). In addition, lots of biologica lclock response element (TCT-motif, ATC-motif, GATA-motif, ATCT-motif, AT1-motif, GT1-motif, MRE, G-Box, G-box, Box-4, Box-II, AE-box, G-box, 1-box, and so on) were also identified.

### Expression Characterization of GhELF4s in Different Tissues

To explore the possible biological functions, the spatio-temporal expression patterns of GhELF4s were investigated in different tissues, including stamen, pistil, petal, root, leaf, stem, and fibers at various developmental stages. According to the existing transcriptional group database of *G. hirsutum*, the FPKM values of the expression of the ELF4 genes were obtained.

**FIGURE 6:**
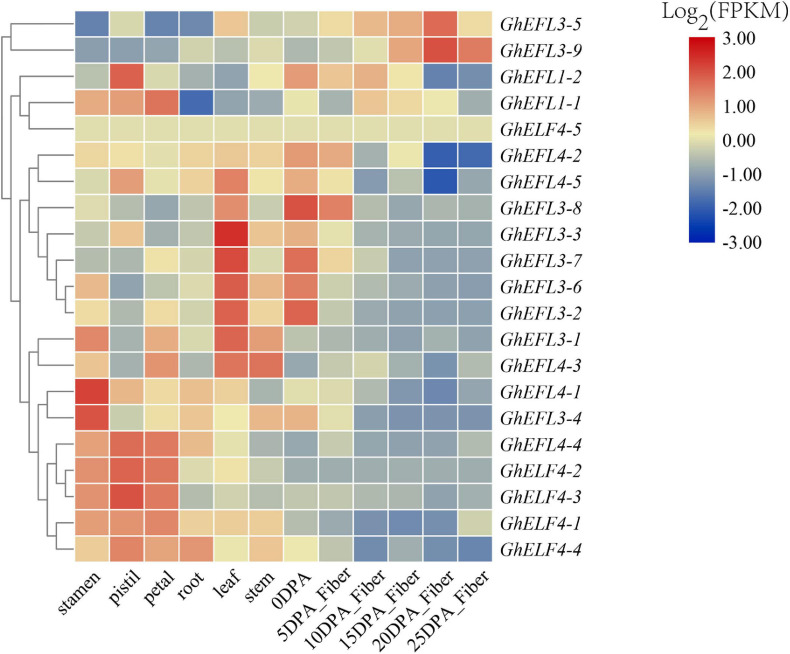
Expression prediction of *GhELF4s* in different tissues. 0 to 25 DPA indicates 0, 5, 10, 15, 20, and 25 days after anthesis. The scale bars in the upper right corner represent log2-transformed FPKM values.

As shown in Figure 6, GhELF4s were most expressed in reproductive organs such as stamens, pistils and petals, followed by leaves, and a few genes are expressed in fibers.

**FIGURE 7:**
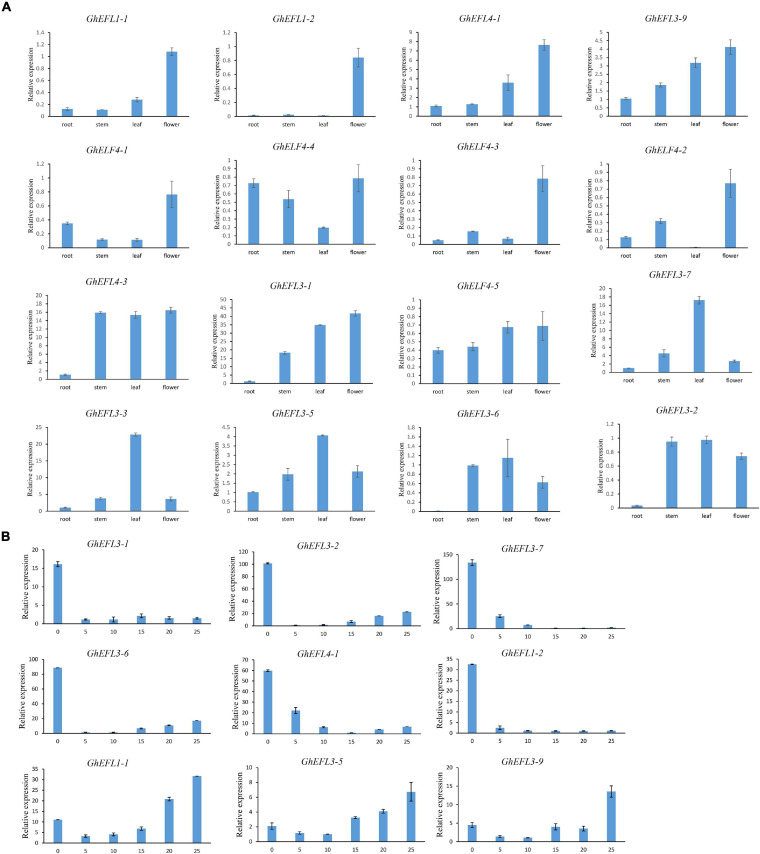
Relative expression levels of GhELF4s in different tissues. **(A)** Expression analysis of GhELF4 in roots, stems, leaves and flowers. **(B)** Expression analysis of GhELF4s in fiber developmental stages. 0, 5, 10, 15, 20, and 25 represent 0, 5, 10, 15, 20, and 25 DPA fibers. Error bars showed the standard deviation of three biological replicates.

On the basis of the transcriptome data, several genes of interest were selected for further verification by qRT-PCR. The primer pairs used in qRT-PCR analysis are detailed in Supplementary Table 5. The gene pairs (*GhEFL1-1*/*GhEFL1-2*, *GhEFL3-2*/*GhEFL3-6*, *GhELF4-1*/*GhELF4-4*, *GhEFL4-2*/*GhEFL4-5*, and *GhEFL3-5*/*GhEFL3-9*) were homologous gene pairs located on At and Dt. The *GhEFL1-1*/*GhEFL1-2*, *GhEFL3-2*/*GhEFL3-6*, and *GhELF4-1*/*GhEFL4-4* gene pairs showed the same expression pattern, and the remaining two homologous gene pairs have different tissue expression patterns. In Figure 7A, most genes were shown to have higher expression levels in flower than in other tissues, except for *GhEFL3-2*, *GhEFL3-3*, *GhEFL3-5*, *GhEFL3-6*, and *GhEFL3-7*. The *GhEFL3-2*, *GhEFL3-3*, *GhEFL3-5*, *GhEFL3-6*, and *GhEFL3-7* genes were highly expressed in the leaf.

### Expression Characterization of GhELF4s in Fiber Development

According to Figure 6 of the transcriptome analysis result, it can be seen that some genes were highly expressed in the fiber. To explore the expression features of *GhELF4s* in fiber development, the expression of nine *GhELF4s* in six stages of fiber development, including 0 DPA, 5 DPA, 10 DPA, 15 DPA, 20 DPA, and 25 DPA stages (Figure 7B).

At initiation stage of the fiber development, the 6 genes (*GhEFL1-2*, *GhEFL4-1*, *GhEFL3-1*, *GhEFL3-2*, *GhEFL3-6*, and *GhEFL3-7*) had the highest expression levels in 0 DPA ovules (Figure 7B). The remaining 3 genes (*GhEFL3-5*, *GhEFL3-9*, and *GhEFL1-1*) exhibited higher levels in 25 DPA fibers than the other 5 stages (Figure 7B).

### The Response of GhELF4s to Abscisic Acid Treatment

**FIGURE 8:**
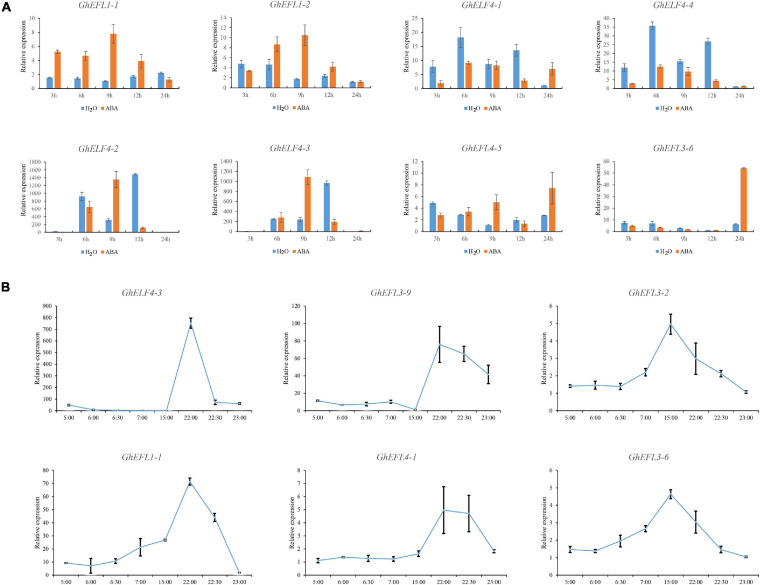
Expression analysis of GhELF4s under ABA and light treatment via qRT-PCR. **(A)** Expression analysis of *GhELF4s* under ABA treatment via qRT-PCR. The TM-1 cotton seedlings were sprayed with water and 100 μM ABA during the two-leaf flattening period, and cotton cotyledon samples were taken at 3, 6, 9, 12, and 24 h. Error bars show the standard deviation of three biological replicates. **(B)** qRT-PCR results of GhELF4s gene response to light at different times. Error bars show the standard deviation of three biological replicates.

The results of the promoter region *cis-*acting element analysis showed that *GhELF4s* might be related to hormone response, and there are more ABA response elements. Therefore, we analyzed the expression characteristics of eight *GhELF4s* under abscisic acid treatment. As shown in Figure 8, *GhELF4-4* and *GhELF4-1*, and *GhEFL1-1* and *GhEFL1-2* were homologous gene pairs located on At and Dt, thus they showed the same expression pattern. Six genes were notably up-regulated, and two genes were notably down-regulated (Figure 8A). The expression trends of *GhEFL1-1* and *GhEFL1-2*, *GhELF4-2*, and *GhELF4-3* were the similar, and they showed high expression up to 9 h of treatment, and after 9 h, the expression began to decrease. The expression patterns of *GhEFL4-5* and *GhEFL3-6* were the similar, both demonstrate high expressions after 24 h of treatment. *GhELF4-4* and *GhELF4-1* were obviously inhibited by ABA.

### Response of GhELF4 Genes to Light in Cotton

ELF4 is a component of the central CCA1/LHY-TOC1 feedback loop in the circadian clock, which is involved in photocycle-sensing and circadian regulation (Doyle et al., 2002). Therefore, we analyzed the expression characteristics of 6 *GhELF4s* under circadian treatment. During the day, from 6:00 to 22:00, there was stable and continuous light in the greenhouse. The greenhouse lights turned on sequence from 6:00 to 6:30, and turned off sequentially from 22:00 to 22:30.

*GhEFL3-6* and *GhEFL3-2* genes were highly expressed at 15:00 (Figure 8B). The expression levels of *GhELF4-3*, *GhELF4-2*, *GhEFL3-9*, and *GhEFL4-1* were highest at 22:00 (Figure 8B), the moment the lights were began to close gradually. *GhELF4s* gene expression changed significantly at dusk. It is speculated that *GhELF4s* are actively expressed in the afternoon. Its were shown that *GhELF4s* expression level reached the maximum value before turning off the light, and *GhELF4s* were sensitive to light and had an obvious response to light regulation.

### Silencing of GhELF4-1 and GhEFL3-6 in Upland Cotton

**FIGURE 9:**
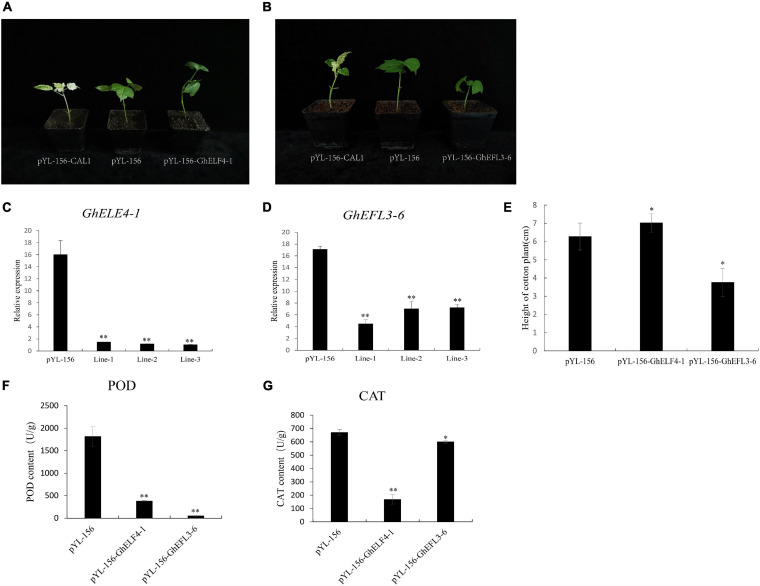
Silencing of *GhELF4-1* and *GhEFL3-6* decreased resistance to salt stress in cotton. **(A)** Comparison of pYL156-GhELF4-1 plant phenotype and pYL-156-cal1, pYL-156; **(B)** Comparison of pYL156-GhEFL3-6 plant phenotype and pYL-156-cal1, pYL-156; **(C)** The relative expression levels of pYL-156 and pYL-156-GhELF4-1; **(D)** The relative expression levels of pYL-156 and pYL-156-GhEFL3-6; **(E)** Plant height statistics of pYL-156, pYL-156-GhELF4-1 and pYL-156-GhEFL3-6; **(F)** VIGS plant Peroxidase (POD) activity assay; **(G)** VIGS plant catalase (Silva et al., 2020) activity assay; **P < 0.01, *P < 0.05.

According to the results of transcriptome analysis, we found that GhELF4 genes are mainly expressed in reproductive organs and in leaves, so we selected two genes with different expression patterns for exploration. *GhEFL3-6* has the highest expression in the leaves, and *GhELF4-1* has a higher expression in the flower, but these two genes have the highest expression in the initial stage of cotton fiber development. Promoter analysis showed that these two genes have the resistance stress *cis-*acting elements MYB and TC-rich repeats. We used VIGS technology to silence the *GhELF4-1* and *GhEFL3-6* genes in the cotton standard line TM-1, respectively, and observe the phenotype of plants. The gene-silenced results showed that the cotton plants of the positive control pYL-156-CAL1 showed albino phenotype (Figures 9A,B). The qRT-PCR results showed that the expression levels of pYL-156-GhELF4-1 and PYL-156-GhEFL3-6 were significantly lower than those of the control plant pYL-156 (Figures 9C,D). After gene silencing of cotton plants, it was found that there were changes in plant height. PYL-156-GhELF4-1 were taller than the control (Figure 9A), pYL-156-GhEFL3-6 were shorter than the control plants (Figure 9B). The statistical results of plant height showed that there was a significant difference in plant height between VIGS plants and control plants (Figure 9E).

To estimate the abiotic stress resistance of the target gene-silenced cotton plants, and the peroxidase (POD) activity and catalase (CAT) (Silva et al., 2020) activity were detected. It was found that the CAT and POD activities in pYL156 cotton plants were significantly higher than those with gene-silenced cotton plants (Figures 9F,G). These results proved that the stress resistance of cotton plants decreased after GhELF4 genes silencing.

## Discussion

### Expansion and Duplication of ELF4 Gene Family

In this study, we performed a comprehensive investigation and analysis of ELF4 genes from four cotton specie. The ELF4s had a close genetic relationship within the same species and with cocoa, and it can also be seen from Figure 2 that the four ELF4 genes of cocoa are equally divided into four groups, and the 12 genes of *G. raimondii* are equally divided into four groups. They are very similar in evolution. According to literature reviews, cotton and cocoa trees came from the same ancestry at least 60 million years ago (Paterson et al., 2012). Cotton contained more ELF4 genes than the species mentioned above (*A. thaliana*, *Populus trichocarpa*, *Glycine max*, *Theobroma cacao*, *Oryza sativa*), indicating that the number of ELF4 genes varies greatly among different species, and the ELF4 genes have been extensively expanded during this period with the evolution of cotton. This indicates that *ELF4s* might evolve in different directions for various species (Paterson et al., 2012). Polyploidization is a major species evolution event, which can provide genetic variation at the whole genome level for the adaptive evolution of plants. *Gossypium* is an allopolyploidized model plant. Gene duplication is a direct result of heterologous polyploidization. Gene duplication events play an important role in the expansion of family genes and the conservation and variation of functions (Paterson et al., 2012; Li and Zhang, 2015). Analysis of gene duplication events in the ELF4s family in cotton revealed that the expansion of ELF4s were mainly due to segment duplication (Zhao et al., 2018; Wu et al., 2019). The ratio of non-synonymous (Ka) substitution to a synonymous (Letunic et al., 2015) substitute ion was used to assess the selection pressure of the homologous genes. All Ka/Ks values in Supplementary Table 3 are less than one. We speculate that the ELF4 gene family has undergone purification selection during the long-term evolution process, leading to segment duplication, and the ELF4s genes may retain their original functions.

### Conservation of ELF4 Genes Sequence and Structure

The ELF4 family was identified based on the DUF1313 domain. DUF1313 is currently only present in ELF4, and the ELF4 family has not been detected outside the plant kingdom (Kolmos et al., 2009). The conservation of sequence structure often indicates the conservation of function. We analyzed the structure and sequence of the ELF4s and the results showed that the gene structure and motif characteristics of the same subfamily were almost the same (Deng et al., 2014). Sequence logo analysis of the conserved domains of four cotton varieties, *Arabidopsis* and *Populus trichocarpa*, as shown in Supplementary Figure 2, DUF1313 (Pfam07011) domain has multiple highly conserved fragments, which are highly conserved in different species. The above analysis shows that the ELF4s family gene sequence structure is highly conserved, so the function of this family genes are also highly conserved.

### Putative Functions of the GhELF4 Gene

Homologous genes often have similar functions, so comparing *GhELF4s* genes with other plant genes will help us analyze the functions of cotton genes.

Previous studies have found that the *AtELF4* plays an important role in the regulation of flowering (Doyle et al., 2002). Overexpression of *AtELF4* transgenic *Arabidopsis* plants showed significant delayed flowering, while *elf4* mutants showed early flowering (Doyle et al., 2002). It was found that the expression of *CCAI* and *LHY* of the *Arabidopsis elf4* mutant decreased, and the expression of *CO* increased, which led to the early flowering of the *elf4* mutant (Doyle et al., 2002; Khanna et al., 2003; Kikis et al., 2005). Overexpression of *GmELF4* transgenic *Arabidopsis* showed delayed flowering, and it was found that the expression of *AtCO* and *AtTF* was reduced (Juliana et al., 2017). Also in recent studies, it was found that the overexpression of *AtEFL1* in elf4 mutants in *Arabidopsis* can not only completely rescue its early flowering phenotype, but also delay flowering (Lin et al., 2019). Overexpression of *AtEFL3* partially rescued the early flowering phenotype of *elf4* mutants (Lin et al., 2019). Overexpression of AtEFL1 and AtEFL3 also inhibited the expression of *AtCO* and *AtTF* (Lin et al., 2019). *AtELF4* is the homologous gene of soybean gene *GmELF4. GhELF4-1*, *GhELF4-2*, *GhELF4-3*, *GhELF4-4*, and *GhELF4-5* in cotton are the homologous genes of *Arabidopsis AtELF4*. *GhEFL3-1*, *GhEFL3-2*, *GhEFL3-3*, *GhEFL3-4*, *GhEFL3-5*, *GhEFL3-6*, *GhEFL3-7*, *GhEFL3-8*, and *GhEFL3-9* are the homologous genes of *Arabidopsis AtEFL3*. *GhEFL4-1*, *GhEFL4-2*, *GhEFL4-3*, *GhELF4-4*, and *GhEFL4-5* are homologous genes of *Arabidopsis AtEFL4*. Comprehensively considering the evolution, result of qRT-PCR and structural conservation of the ELF4 gene family, as well as the similarity of gene sequences, we speculate that these cotton genes may have a conserved function with *AtELF4*, *AtEFL3*, *AtEFL4*, and *GmELF4*, and may play an important role in flowering regulation.

*ELF4* is a circadian rhythm gene related to light regulation and light processes in some GhELF4s in upland cotton. The qRT-PCR results found that the gene family was most actively expressed in the afternoon, which is consistent with previous research results (Doyle et al., 2002), and there seem to be two expression patterns, one is the highest expression at 22:00, the other is the highest expression at 15:00. The results indicate that the GhELF4s play a role in the light pathway and light can affect the expression of GhELF4s function. *GhELF4-1*, *GhELF4-2*, *GhELF4-3*, *GhELF4-4* and *GhELF4-5* are a homologous gene of *AtELF4* and also the most studied ELF4-family gene in *Arabidopsis*. The study found that ELF4 is a negative feedback loop of the phytochrome regulator and is related to the photoperiod (Kikis et al., 2005). The *Arabidopsis elf4* mutant has an impaired ability to perceive sunlight, and the circadian rhythm is unbalanced, indicating that ELF4 is related to light input (Doyle et al., 2002). Moreover, studies have found that ELF4 transcripts maintain a steady rhythm under continuous light, which shows that transcript abundance is under circadian control, and robust cycling is not detectable after transfer to continuous darkness (Doyle et al., 2002). Previous studies also found that *ELF4* is a gene necessary for the initial light-induced expression of *CCA1* and *LHY* mediated by plant pigments and the subsequent light-induced circadian oscillations, which also highlights the importance of *ELF4* in mediating the light input of the central oscillator (Kikis et al., 2005; Kim et al., 2012, 2013). *GhELF4-1*, *GhELF4-2*, *GhELF4-3*, *GhELF4-4*, and *GhELF4-5* as the homolog of the *AtELF4* gene, and its specific function, needs to be further studied in cotton.

According to the prediction of the above-mentioned homologous genes, combined with the transcriptome analysis data Figure 6 and qRT-PCR results Figure 7A, all these indicated that GhELF4s might play an important role in regulating flowering and light pathways.

Studies have found that ELF4s also play an important role in the development of cotton fibers. *GhEFL3-5*, *GhEFL3-9*, and *GhEFL1-1* are up-regulated with fiber development at 20–25 DPA which is the secondary wall thickening period (Figure 7B). *GhEFL4-1, GhEFL1-2, GhEFL3-1, GhEFL3-2, GhEFL3-6*, and *GhEFL3-7* expressed the highest in the initial stage of fiber development, which may be related to the initiation of fiber development. *GhEFL3-1*, *GhEFL4-1*, and *GhEFL4-4* is the homologous gene of *Doritaenopsis hybrid* gene *DhEFL2*, *DhEFL3*, and *DhEFL4*, respectively. *DhEFL2*, *3*, or *4* could regulate hypocotyl formation. And they found that the length of the *elf4-1* mutant’s hypocotyl was longer than that of wild-type *Arabidopsis* (Silva et al., 2020). In *Arabidopsis*, the complex EC composed of ELF3, ELF4 and LUX can regulate the elongation of hypocotyls in the early evening, which may be related to cell differentiation and elongation. Therefore, it is speculated that the *GhELF4-2*, which is homologous to *AtELF4*, also has similar functions. These indicate that GhELF4 family plays an important role in regulating cotton fiber development.

ABA is a plant hormone that inhibits growth. It can promote leaf shedding, make buds enter a dormant state, inhibit cell elongation, promote fruit maturity and respond to abiotic stress. Previous studies have shown that ABA regulates flowering by affecting the photoperiod, and plant pigment A can promote the degradation of ABA (Arve et al., 2013; Wang et al., 2013). In this study, on the basis of the transcriptome data and the qRT-PCR results, the expression of *GhEFL1-1* and *GhEF1-2* changed significantly during ABA treatment and light treatment, and these have higher expression levels in the flower (Figure 7A), which may be related to the ABA pathway and photoperiod in upland cotton (Figure 8).

Specific fragment of two GhELF4 were identified for gene silencing (Supplementary Figure S3). After *GhELF4-1* and *GhEFL3-6* were silenced, the height of VIGS seedlings was significantly different. When the *GhEFL3-6* gene was silenced, cotton seedlings became dwarf. ELF4 is involved in photoperiod perception and circadian regulation (Doyle et al., 2002; Khanna et al., 2003; Kikis et al., 2005), and deletion of ELF4 can cause circadian clock disturbance, photoperiod perception is affected (Doyle et al., 2002). The qRT-PCR results also showed that *GhEFL3-6* was highly expressed in leaves (Figure 7A). And leaves are the most effective part of the photoperiod perception (Song et al., 2015), we speculate that the silence of *GhEFL3-6* could affect the perception of photoperiod by leaves, but this speculation need further proof. CAT is one of the antioxidant enzymes of plants. It can resist some biological and non-biological damages from the outside world, and can maintain the normal operation of plant cells. POD enzyme can detoxify, catalyze toxic substances in cells, and protect cells. Both POD and CAT are enzymes related to plant resistance stress. The CAT and POD enzyme activities in VIGS cotton plants were lower than the control, indicating that these two genes were related to the maintenance of normal cell development, and resistance decreased after gene silencing. How *GhELF4-1* and *GhEFL3-6* affect the height of cotton plants and the degree of cotton resistance need to be further explored.

## Conclusion

This study systematically examined the gene structure, physical and chemical properties, protein domains, Mw, pI, subcellular localization, gene expression, phylogeny, and collinearity of ELF4s. Genes of the ELF4 family are essential for plant growth, development, and flowering. ELF4s gene were identified in *A. thaliana*, *Populus trichocarpa*, *Glycine max*, *Theobroma cacao*, *Oryza sativa*, and four cotton species to construct a phylogenetic tree. At the same time, according to chromosomal location and gene duplication events, ELF4 genes were divided into four subfamilies. The results of qRT-PCR showed that the expression of the *GhELF4* gene in different organs is different, mainly in the flowers and leaves. The analysis of the promoter showed that GhELF4s function in the ABA pathway. The light response experiment proved that the GhELF4s also plays an important role in the light response pathway. The silencing of *GhELF4-1* and *GhEFL3-6* had a significant effect on the plant height of cotton seedlings. And the resistance of cotton seedlings decreased after gene silencing. The results provide a basis to further explore the function of ELF4 in cotton.

## Data Availability Statement

The original contributions presented in the study are included in the article/Supplementary Material, further inquiries can be directed to the corresponding author/s.

## Author Contributions

HWa: conceptualization. XY: data curation. JZ: formal analysis and software. SY and HWa: funding acquisition and writing – review and editing. MT, AW, JZ, HWe, MZ, JL, and XF: methodology. MT: validation, visualization, and writing – original draft. All authors contributed to the article and approved the submitted version.

## Conflict of Interest

The authors declare that the research was conducted in the absence of any commercial or financial relationships that could be construed as a potential conflict of interest.

## Supplementary Material

Supplementary Figure 1 Analysis of conserved domains on ELF4s family proteins in cotton.

Supplementary Figure 2 Sequence logos of the conserved domain DUF1313 in *G. arboreum*, *G. raimondii*, *G. hirsutum*, *G. barbadense*, *Arabidopsis*, and *Populus trichocarpa*.

Supplementary Figure 3 Silencing fragment of GhELF4 genes.

Supplementary Table 1 Physicochemical characteristics of ELF4 proteins of *A. thaliana*, *Populus trichocarpa*, *Glycine max*, *Theobroma cacao*, and *Oryza sativa*, *G. arboreum*, *G. raimondii*, *G. hirsutum*, and *G. barbadense*. Including amino acid length, protein isoelectric point, protein molecular weight, hydrophilicity and subcellular location.

Supplementary Table 2 Gene locus information of ELF4s in *G. arboreum*, *G. raimondii*, *G. hirsutum*, and *G. barbadense*.

Supplementary Table 3 The homoeologous gene pairs information in *G. hirsutum*, *G. raimondii*, and *G. arboretum*.

Supplementary Table 4 Positions of the conserved domains of *G. arboreum*, *G. raimondii*, *G. hirsutum* and *G. barbadense*.

Supplementary Table 5 Primer pairs used in the qRT-PCR analysis.
