# Supplementary figures and images for: Genome-Wide Identification of the Early Flowering 4 (ELF4) Gene Family in Cotton and Silent GhELF4-1 and GhEFL3-6 Decreased Cotton Stress Resistance
Source: Front Genet. 2021 Jul 6;12:686852. doi: 10.3389/fgene.2021.686852 (PMC8315153; doi:10.3389/fgene.2021.686852)

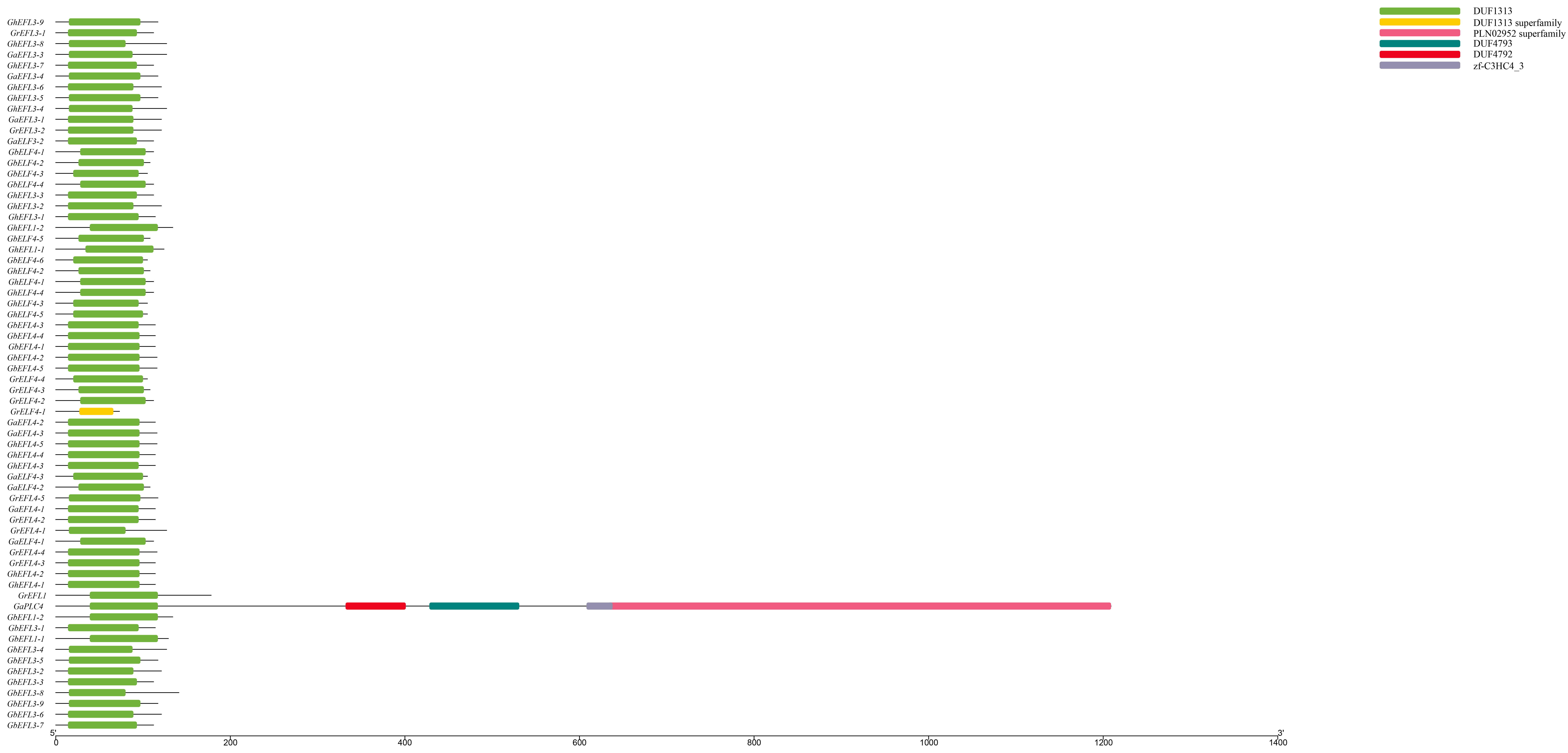

Supplement: Supplementary Figure 1 — Analysis of conserved domains on ELF4s family proteins in cotton. [file Image_1.TIF]

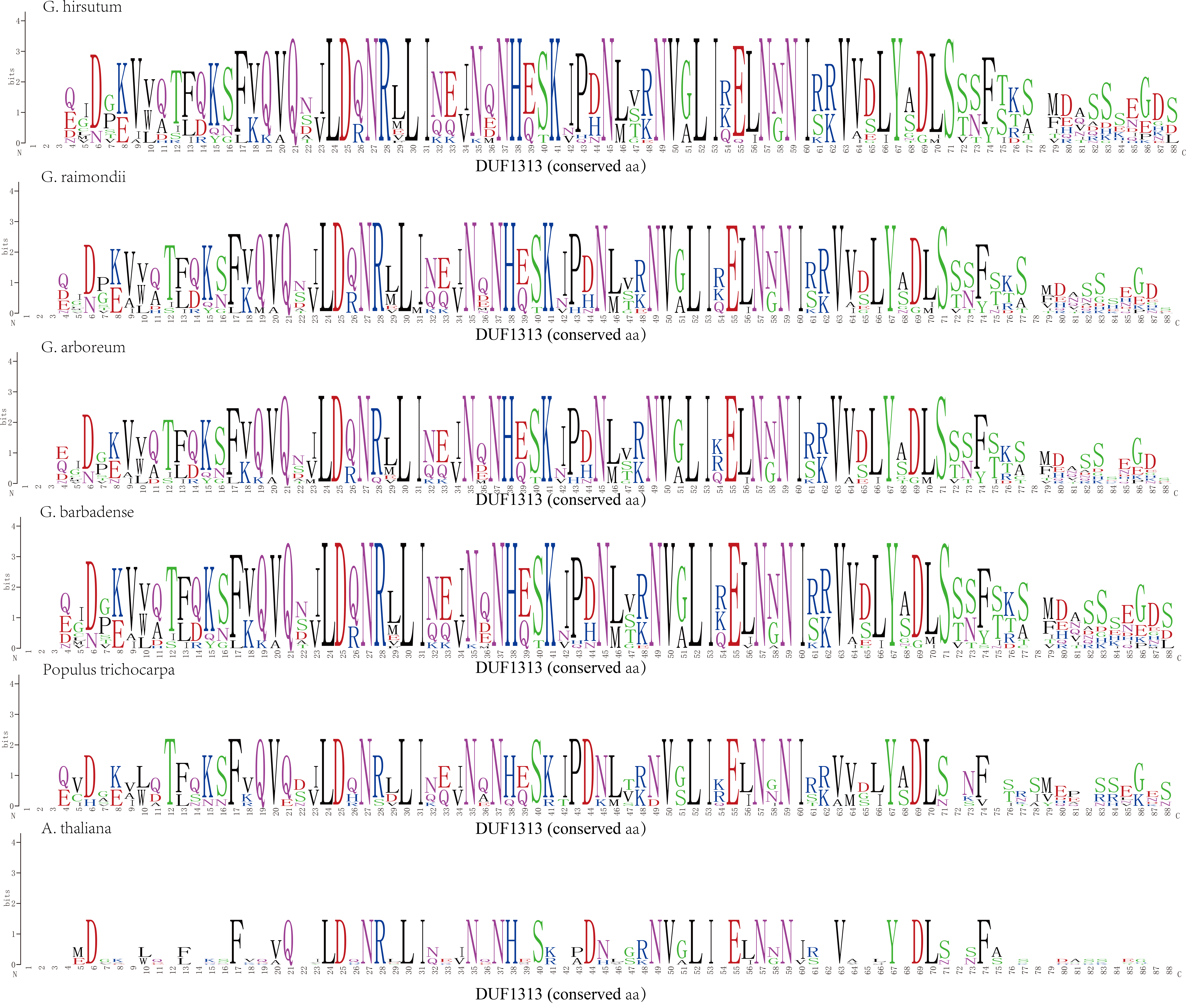

Supplement: Supplementary Figure 2 — Sequence logos of the conserved domain DUF1313 in G. arboreum, G. raimondii, G. hirsutum, G. barbadense, Arabidopsis, and Populus trichocarpa. [file Image_2.TIF]

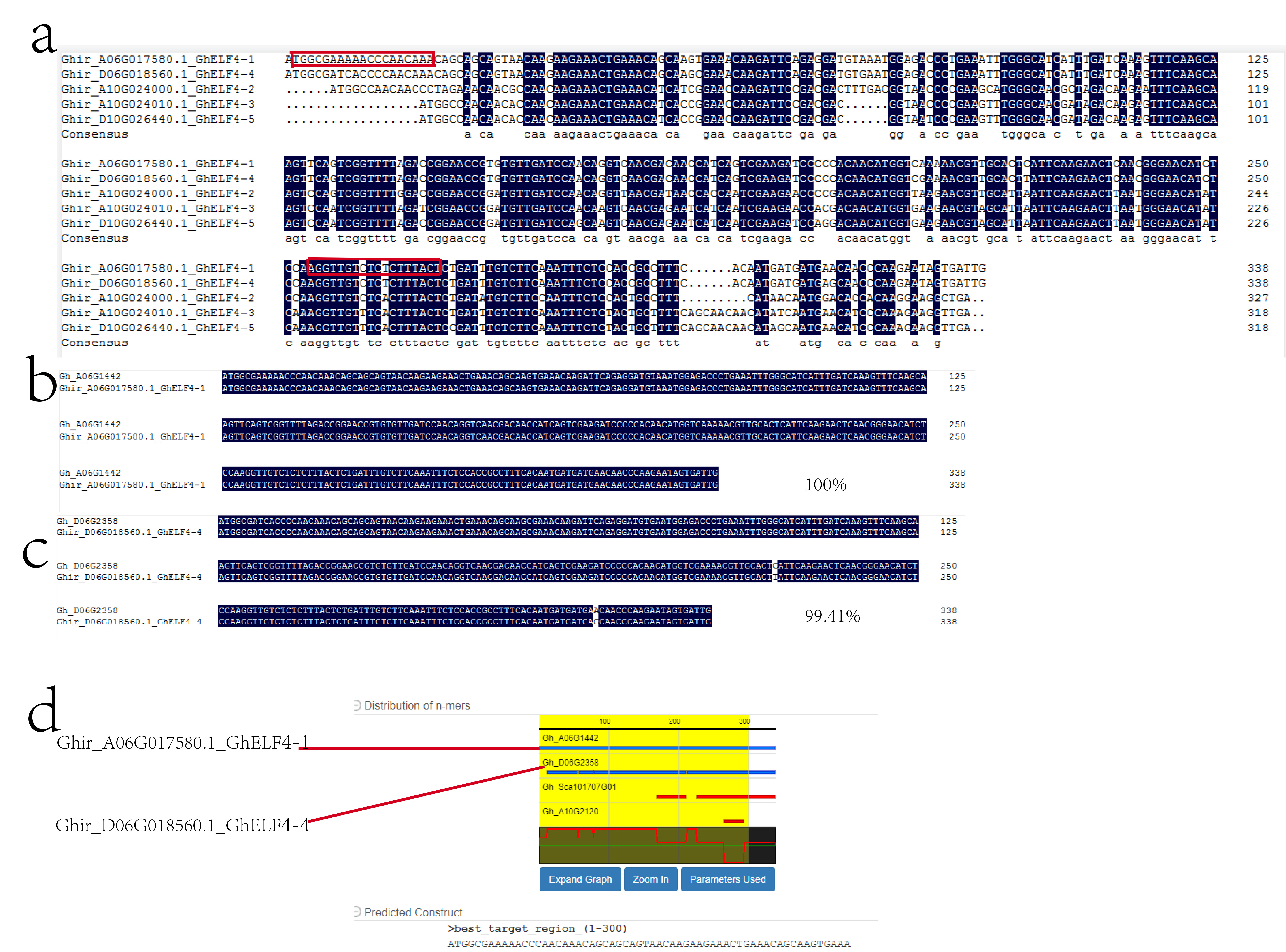

Supplement: Supplementary Figure 3 — Silencing fragment of GhELF4 genes. [file Image_3.JPEG]
